# 1271. Antimicrobial Appropriateness after Ambulatory Antimicrobial Stewardship Program for Uncomplicated Cystitis

**DOI:** 10.1093/ofid/ofad500.1111

**Published:** 2023-11-27

**Authors:** Harit Thongwitokomarn, Saowaluck Yasri, Romanee Chaiwarith

**Affiliations:** Faculty of medicineChiang Mai University, Chiang Mai, Chiang Mai, Thailand; Faculty of Medicine, Chiang Mai University, Thailand, Muang, Chiang Mai, Thailand; Faculty of Medicine, Chiang Mai University, Chiang Mai, Chiang Mai, Thailand

## Abstract

**Background:**

Urinary tract infection (UTI) is a common bacterial infection found in outpatient departments. Previous studies showed the rising trend of fluoroquinolone use for urinary tract infections treatment coinciding with the increase of fluoroquinolone-resistance uropathogens. The major cause of this problem is inappropriate antimicrobial use. Therefore, we implemented antimicrobial stewardship programs to improve antimicrobial appropriateness and treatment outcome in ambulatory uncomplicated cystitis patients at Maharaj Nakorn Chiang Mai Hospital.

**Methods:**

We conducted a retrospective pre-post intervention study design among ambulatory uncomplicated cystitis patients aged ≥ 18 years in Maharaj Nakorn Chiang Mai Hospital compared the pre-intervention group (1 March 2021 – 31 August 2021) and post-intervention group (1 October 2021 – 31 March 2022).

**Results:**

There were 49 patients included in the study; 29 were in the pre-intervention group and 20 were in the post-intervention group. Antimicrobial appropriateness increased significantly (0% vs 55%, P< 0.001) after antimicrobial stewardship programs’ implementation attributed to significant improvement in appropriate antimicrobial choice (6.9% vs 95%, P< 0.001), dose (0% vs 89.5%, P= 0.029) and duration (6.9% vs 95%, P< 0.001). The antimicrobials were indicated in post-intervention group more than pre-intervention group but did not reach statistically significance (44.8% vs 65%, P=0.164). There was a significant improvement in concordant antimicrobial therapy after the antimicrobial stewardship programs’ implementation (25% vs 92.3%, P= 0.001), although there was no difference in treatment failure between groups (12.5% vs 7.1%, P= 1.000).

**Figure:**
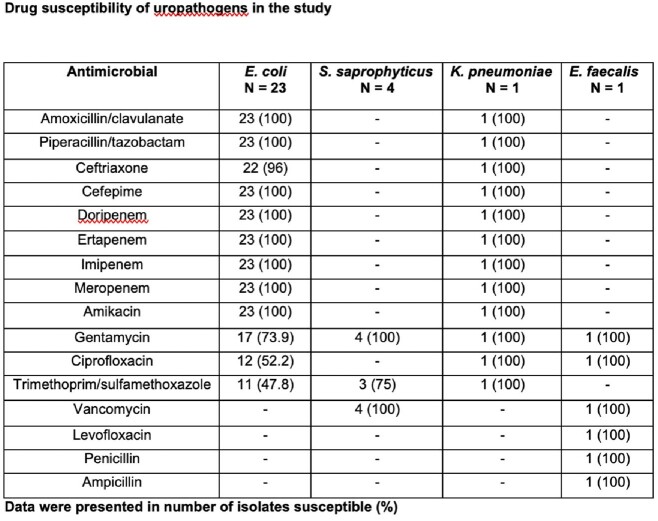

**Figure:**
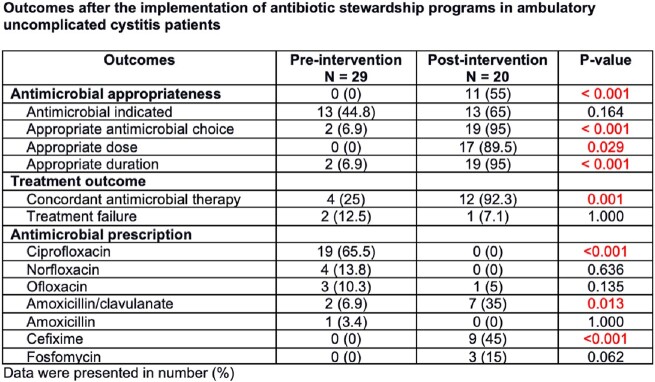

**Conclusion:**

Antimicrobial appropriateness and concordant antimicrobial therapy increased significantly after antimicrobialstewardship programs’ implementation. However, there was no difference in treatment failure between groups.

**Disclosures:**

**All Authors**: No reported disclosures

